# Silence, power and communication in the operating room

**DOI:** 10.1111/j.1365-2648.2009.04994.x

**Published:** 2009-07

**Authors:** Fauzia Gardezi, Lorelei Lingard, Sherry Espin, Sarah Whyte, Beverley Orser, G Ross Baker

**Affiliations:** 1 Fauzia Gardezi MA Research Associate The Learning Institute, The Hospital for Sick ChildrenToronto, Canada; 2 Lorelei Lingard PhD Lead Educational Researcher The Learning Institute, The Hospital for Sick Children, and Associate Professor Department of Pediatrics and University of TorontoCanada; 3 Sherry Espin RN PhD Associate Professor Ryerson University, Daphne Cockwell School of NursingToronto, Canada; 4 Sarah Whyte MA PhD Candidate and Research Associate The Wilson Centre for Research in Education, University of TorontoCanada; 5 Dr Beverley Orser MD PhD FRCPC Professor of Anesthesia and Physiology Canada Research Chair in Anesthesia, University of Toronto and Department of Anesthesia, Sunnybrook Health Sciences CentreCanada; 6 G. Ross Baker PhD Professor Department of Health Policy, Management and Evaluation, University of TorontoCanada

**Keywords:** communicating, ethnography, nurse–physician relationships, operating room, power, silence, theatre nursing

## Abstract

**Title. Silence, power and communication in the operating room:**

**Aim**

This paper is a report of a study conducted to explore whether a 1- to 3-minute preoperative interprofessional team briefing with a structured checklist was an effective way to support communication in the operating room.

**Background**

Previous research suggests that nurses often feel constrained in their ability to communicate with physicians. Previous research on silence and power suggests that silence is not only a reflection of powerlessness or passivity, and that silence and speech are not opposites, but closely interrelated.

**Methods**

We conducted a retrospective study of silences observed in communication between nurses and surgeons in a multi-site observational study of interprofessional communication in the operating room. Over 700 surgical procedures were observed from 2005–2007. Instances of communication characterized by unresolved or unarticulated issues were identified in field notes and analysed from a critical ethnography perspective.

**Findings**

We identified three forms of recurring ‘silences’: absence of communication; not responding to queries or requests; and speaking quietly. These silences may be defensive or strategic, and they may be influenced by larger institutional and structural power dynamics as well as by the immediate situational context.

**Conclusions**

There is no single answer to the question of why ‘nobody said anything’. Exploring silences in relation to power suggests that there are multiple and complex ways that constrained communication is produced in the operating room, which are essential to understand in order to improve interprofessional communication and collaboration.

**What is already known about this topic**

- Nurses often feel constrained in what they are able to say in the operating room, or feel that they are a passive audience for others.
- Many silences in the operating room derive from fear of appearing inadequate or incompetent in front of other operating room professions.
- Theories of silence and power suggest that silence is not a straightforward reflection of powerlessness; it may also be used strategically, for example, as a means of exerting power or resisting power.

**What this paper adds**

- There are multiple forms of problematic silences in the operating room, including the absence of communication, non-response to a colleague’s question or request, and quiet or hesitant speech.
- Silence may reflect powerlessness, but at times may also be a form of expression used by nurses and other operating room professionals to accomplish objectives.
- As well as reflecting individual behaviours, silences may reflect predispositions or internalized factors resulting from broader institutionalized power relations.

**Implications for practice and/or policy**

- Policies to promote safety in the operating room by encouraging team members to ‘speak up’ are important but cannot ignore how speech and silence interact and shape each other.
- How we approach and train for interprofessional collaboration should incorporate an awareness of the complex strategies and modalities of communication, including silence, employed in the operating room setting.

## Introduction

Research suggests that inadequate communication is a primary cause of medical errors and that communication among the professions in the operating room (OR) is essential to patient safety (Gawande *et al.* 2003, Sutcliffe *et al.* 2004, Gandhi 2005, Joint Commission on Accreditation of Healthcare Organizations 2008). In research on nurse–physician communication in settings such as ORs or ward rounds, nurses persistently report that they are perceived as a passive audience for others, and that they are constrained in what and when they are able to communicate (Manias & Street 2001, Lingard *et al.* 2004). The communicative constraints on nurses have been analysed in terms of the ways that knowledge and competence are displayed in the ‘theatre’ of the OR (Riley & Manias 2005, Gillespie *et al.* 2007), the continued dominance of bio-medical discourse over other types of healthcare discourse (Björnsdottir 2001, Coombs 2003), and the disempowered or ‘oppressed group’ status of nurses (Kuokkanen & Leino-Kilpi 2000, Bradbury-Jones *et al.* 2008). Nurses also report seeing themselves as ‘keepers of the peace’ whose role is to maintain a calm environment for surgeons to focus on their work, sometimes described as a gendered role or a ‘female thing’ (Riley & Manias 2005).

Survey research on team communication in the OR indicates that nurses and anaesthesiologists have less positive perceptions of the effectiveness of their communication compared with surgeons, and are less likely to respond positively to the statement ‘I am comfortable intervening in a procedure if I have concerns about what is occurring’ (Mills *et al.* 2008). In our own ethnography, leaders of the different professions spoke to us about occasions when something of concern took place in the OR and ‘nobody said anything’. Because of their central role in patient safety and advocacy, nurses are often the subject denoted in questions about why no one spoke up.

To date, there has been no research directly examining the speech practices, including silence, that are identified as constrained or problematic. This is understandable given the difficulty of documenting silences in communication and the traditionally marginal role of silence in qualitative research (Poland & Pederson 1998). Using observational data from a multi-year study of interprofessional communication in three hospital ORs, our objective in this paper is to directly examine instances of silence and constraint in communicative exchanges in the OR using a critical ethnography approach.

Critical theory represents a wide-ranging tradition that shares a critique of societal institutions and an invested approach to research for the purposes of positive action or change. Critical ethnography differs from conventional ethnography in that it includes a focus on social structures: ‘in addition to portraying their informants’ world view, critical theorists also aim to reveal socioeconomic conditions that produce and reinforce asymmetrical structures of control’ (Jermier 1998, p. 240). Two specific features of a critical approach inform our ethnography of silence: (1) attention to power dynamics in silence and (2) the usefulness of critical methodology for analysing silences.

## Background

### On silence and power

While speech is often equated with an active stance of self-determination and self-expression, silence is typically viewed as an indication of self-censorship, passivity or quiescence. Therefore, explicit attention to silence may be seen as a way of attending to the voices of those with less power. To the extent that silence is revealing of dynamics of power and privilege, it is important to ‘listen’ to silence (Mazzei 2007).

Yet silence is not a straightforward reflection of powerlessness, nor is speech a straightforward reflection of power. Researchers in the fields of sociolinguists and feminist anthropology have explored the strategic use of silence. Glenn (2004) suggests that disadvantaged groups often employ ‘a rhetoric of silence’ as a means of subverting power. Similarly, Gal (1991) details research on varied forms of cultural expression adopted by women – genres of communication that are at times veiled, ambiguous, laconic or indirect – which, on the surface, may be perceived as silent and inarticulate, but which may also be ways of asserting one’s own power or resisting that of another.

Poststructural approaches also put forward a view of silence as potentially strategic (Mazzei 2007) and challenge the uniform valorization of ‘voice’. Brown (2005) contends that, in many cases, speech is not a reflection of ‘authentic voice’ but of the role of discourse in producing disciplined subjects. She gives the example of the tell-all, confessional discourse pervading modern western culture; not participating in this discourse by adopting a stance of silence may afford a measure of freedom. This is not to say that discursive projects do not create silences and silencing processes, but silences may also ‘function as that which discourse has not penetrated, as a scene of practices that escape the regulatory functions of discourse’ (Brown 2005, p. 88).

Thus, in terms of their ability to communicate, or as modes of expression, neither silence nor speech is straightforwardly negative or positive. Silence may be a means of exerting power over others, a reflection of relative powerlessness or a means of resisting power. Speech is a means of self-expression, but may also be used to silence others or may reflect a lack of individual agency if it takes the form of participation in regulatory or normative discursive projects.

These complexities underline the importance of examining the context of communication – not just the immediate local and individual context, but larger institutional, cultural and structural contexts – to understand the meaning of silence (Gal 1991, Poland & Pederson 1998). They also reflect the close interplay between speech and silence. Brown (2005) emphasizes that silence and speech are not opposites, but organize and co-create each other. Mazzei (2007) suggests a view of silence that ‘places it not in opposition to speech, but that positions silent speech on a continuum with voiced speech’ (p. 633). Similarly, we investigate that which remains unresolved or unarticulated in OR communication by examining the interplay between speech and silence. Furthermore, we suggest that silence is reflective of power dynamics and can help in understanding when, where and why communication is constrained.

### Observing, recording and interpreting silence

A critical ethnography approach is useful in understanding phenomena such as silence and constraint in communication, which are ambiguous and difficult to record and interpret ‘objectively’. First of all, in contrast to the idea that one can produce an objective record of field observations, a critical approach emphasizes that observations and records of those observations are filtered and mediated through our own imperfect senses and ‘tacit presuppositions’ (Bourdieu 1996). Furthermore, a central characteristic of communication is that meanings are not unequivocal; they are ambiguous and indeterminate both in how they are delivered and how they are received. From a critical perspective, we understand our field notes and observational interpretations to be ‘texts’. Following Foley (2002, p. 473), we seek ‘provisionally accurate’ interpretations, for we ‘understand that writing is inscription, an evocative act of creation and of representation’ (Denzin 1997, p. 25–26). Finally, part of the self-awareness needed on the part of the ethnographer is that one’s position as an outsider in the research setting, requiring a roadmap to understand what one is seeing, can lead to a privileging of the empirically overt, such as that which is spoken, over underlying social relations (Bourdieu 1977). A critical approach to silence emphasizes an awareness of the interplay between social structures and local context.

## The study

### Aim

The aim of the study was to explore whether a 1- to 3-minute preoperative interprofessional team briefing with a structured checklist was an effective way to support communication in the OR.

### Design

This was a retrospective study. We did not set out to record silences in OR communication. The focus of the research was the collection and analysis of data on interprofessional communication about the patient and the surgical procedure. However, various forms of silence, particularly the unresolved and unarticulated, were one of the most evident forms of interprofessional communication we observed, and provided the impetus for this paper. In this paper, we report on data gathered as part of a multi-site study of interprofessional communication in the OR.

### Setting

The research was undertaken in general surgery at three tertiary-care hospitals in Toronto, Canada. Two of the sites were large urban academic teaching hospitals and the third was a smaller combined teaching and community hospital.

### Participants

Participants in the study were 11 general surgeons and all members of OR teams working in those surgeons’ ORs, including 116 OR nurses and 74 anaesthesiologists. OR teams were typically comprised of a surgeon, a scrub nurse, one to two circulating nurses, an anaesthesiologist or anaesthesia fellow, and two to three surgical trainees. Nursing and anaesthesia trainees, and respiratory therapists, were periodically present as well. There was a significant degree of rotating membership on the teams. For example, most nurses were not assigned to work with the same surgeon all the time, and anaesthesiologists worked in multiple services. Thus, the OR teams we observed were characterized by a core of general surgeons, with a larger core of nurses who worked in general surgery, anaesthesiologists who worked in multiple services, trainees on rotation, occasional nursing staff from other services and sometimes anaesthesiologists from other hospitals.

### Data collection

We collected observational data, using principles of ethnographic research. Observers were present in the OR and recorded notes on interprofessional communication about the patient and procedure. Notes were recorded during the surgical procedure, and elaborated and reflective field notes were produced after the sessions ended. Standard ethnographic techniques for writing field notes were used (Hammersley & Atkinson 2007). Data were collected between August 2005 and December 2007, and just over 700 surgical procedures were observed.

### Ethical considerations

The study was approved by the appropriate research ethics boards. We conducted information sessions with OR staff before beginning our research to explain the study and distribute consent forms. Observers continued to obtain signed consent from OR team members as the study progressed. If OR team members did not consent to observations, we did not record observations of interactions involving that OR team member.

### Data analysis

The data presented here are taken from instances of interprofessional communication in field notes that were coded by one of four trained observers as being characterized by unresolved or unarticulated issues. This coding was done for all field notes and reviewed for consistency of coding by at least two other researchers. One researcher (FG) further analysed these instances categorizing them into three predominant forms of silence. Most of these coded instances involved silences in nurse–physician communication. In our data set, anaesthesiologists tended to talk and interact less overall, so that we had fewer opportunities to capture their communication or to characterize their silences by references to contextualizing speech.

## Findings and discussion

Silence and quiet can play useful roles in the OR, and are necessary for the safe performance of some tasks. At times, silence may reflect an OR team that has an experienced, familiar and comfortable working relationship. The focus here is on constrained communication: on why an OR professional may remain silent when something of concern takes place. The instances we examine do not all relate to issues of patient safety; many are much more mundane exchanges. Yet, power dynamics often reveal themselves in communication over mundane and routine matters.

To investigate silence, one is not only examining silence, but also speech and the interplay between speech and silence. This view of silence is reflected in the three forms of silence discussed below: (1) absence of communication, made evident by prior actions or communication; (2) lack of response to a direct address by another, or responding with silence to another’s question or directive; and (3) aspects of delivery that blur the lines between speech and silence, such as speaking quietly, timidly or hesitantly. We give examples of these and discuss them in terms of dynamics of power within interprofessional communication.

### Absence of communication

Absence of communication is ‘observable’ when it can be deduced from situational factors. For example, it can reveal itself when staff do not seek clarification, ask follow-up questions, or communicate immediately relevant information. Consider the following example, in which the nurse does not fulfil the surgeon’s request because she is uncertain which controls to use. She does not verbalize this uncertainty, even after two requests for her to perform an action:

Surgeon asks for insufflation of the patient’s abdomen. The circulating nurse says, ‘All right’ but does not take any action. A few seconds later the request is repeated. The nurse still does not act. The surgeon notices the nurse standing in the middle of the room looking uncertain. The surgeon tells the nurse which controls to use. She goes to the controls and adjusts them.

Fear of exposing a lack of knowledge is one possible motivation for some silences observed in the OR. Using Goffman’s (1969) theatrical metaphors, Riley and Manias (2005) discuss this phenomenon in terms of shifts between ‘front’ and ‘back stage’ behaviour. Back stage is a physical or temporal space in which one is more relaxed and less attuned to exhibiting normative behaviour for an implied audience, whereas front stage is a space of managed public performance. Riley and Manias describe the effort nurses will often put into maintaining an outward appearance of competence in the OR as ‘front stage’ behaviour for a surgeon audience. Meanwhile, nurses may engage in ‘back stage’ behaviour, such as consulting with each other in hushed voices about a surgeon’s preferences, to avoid publicly appearing uncertain.

We observed something similar in terms of nurses often speaking to other nurses when trying to resolve a problem rather than approaching surgeons, even when the issue was one that could be resolved more directly by asking a member of the surgical team. The nurses in the following excerpt appear uncertain about how to act on a surgical request, but also hesitate to voice the need for clarification:

A surgical resident asks for the cautery to be set at ‘60 spray mode’. A circulating nurse changes the settings.

The resident asks again for ‘spray mode’.

The circulating and scrub nurses examine the cautery machine. No information is relayed to the surgical team.

Two minutes later the resident asks again if the cautery is on spray mode.

The scrub nurse says ‘There is none’.

A second surgical resident says, ‘There always is’.

The surgical fellow inspects the machine and points to the spray mode adjustment. The circulating nurse adjusts it to spray mode.

After the first two requests by the surgical resident, the nurses say nothing about being unable to locate the ‘spray mode’. They consult each other, but do not report their uncertainty to, or seek clarification from, the surgeons. They may have been inclined not to bother the surgeons, but they may also have perceived this to be within their scope of practice and thus did not want to ask in order to save face. As Riley and Manias (2005) suggest, concern about betraying a lack of knowledge may encourage a self-protective silence; being reprimanded for not knowing a surgeon’s preferences, for example, was a common experience for nurses in their study. These examples illustrate how power and status hierarchies come into play in seemingly mundane communication surrounding the completion of routine tasks.

Absence of communication is also sometimes evident even when there is no potential for revealing a lack of knowledge. Examples include not sharing information that others do not possess or not providing follow-up communication. In one example, the surgeon enters the OR, greets the patient and asks if he is nervous. A nurse says quietly, to herself, ‘No speak English’, but does not inform the surgeon that the patient does not understand what he is saying. In another example, a surgeon requests a different light source because the one that is set up is not providing sufficient light to perform the laparoscopic surgery. A nurse brings a new light source into the room but does not ask if it should be set up. She leaves the room and several minutes later the surgeon asks if the light source has arrived. The absence of follow-up communication suggests a general reticence in nurses’ communication with physicians.

Our observations suggest that a reticent tone and form of speech are at times tactical, a strategic mode of speech that nurses adopt. We observed nurses using a laconic style to influence the behaviour of others, to chastise, or to encourage events in the OR to flow the way they wanted them to. In the example below, the nurse uses a combination of minimal words and direct action to obtain a response from a resident who is putting off her requests for information:

After the patient has arrived in the OR, the anesthesiologist asks the surgical resident if the surgical team will want the patient’s arms to be tucked in for the surgery.

Surgical resident says he does not know, but will ask, and then leaves the room.

Circulating nurse: ‘Arms out?’

Anesthesiologist: ‘He said he didn’t know’.

After the patient is anesthetized the surgical resident returns to the room and begins catheter insertion. He does not report back about arm positioning.

Circulating nurse: ‘So the arms can stay out?’

Surgical resident: ‘I’ll ask [surgeon]. I’m not sure’.

Surgical resident continues working on the patient.

A minute later circulating nurse asks ‘He’s [surgeon’s] not here?’

Surgical resident: ‘He’s in trouble?’

Nurse does not say anything but goes to the phone.

Resident looks at nurse and says ‘Hm?’

No answer from nurse. Nurse says into phone, ‘Can you page [surgeon], please’.

A few seconds later the phone rings. Nurse answers and tells resident to come to the phone. Nurse holds receiver to resident’s ear because he is wearing gloves and holding prep solution. Resident asks surgeon about type of incision and then arm positioning.

The resident says twice that he will ask the surgeon for the information the nurse wants, but appears to be in no hurry to do so. The nurse asks if the surgeon is not in the OR, perhaps to clarify why the resident is not asking the surgeon about arm positioning. The resident’s response (‘He’s in trouble?’) sounds somewhat provocative, a little as if he is saying, ‘What’s the problem?’ The nurse does not respond; yet, with minimal further communication, she manages to have him ask the surgeon for the information she wants. This strategy was characteristic of at least two nursing team leaders who were present for multiple observation sessions.

### Not responding to queries or requests

The silence we observed in the OR often took the form of non-responses to direct questions or requests. Non-responses may relate to not hearing the address or to mental preoccupation with a task at hand. What we examine here are instances of non-response that do not appear to be entirely attributable to such factors. Purposeful silences of this sort can be difficult to identify, and our recognition of them often relates to the number of times a question is asked. The following event contains several unanswered queries by nurses to surgeons:

After the surgery has commenced, the circulating nurse asks the surgeons twice for the preoperative diagnosis but gets no answer.

Six minutes later, when the anesthesiologist happens to walk past, the circulating nurse asks, ‘Dr. [name], did you say that this patient has Crohn’s?’

Anesthesiologist: ‘No, she has ulcerative colitis. Well, they’ve taken out her colon so technically she doesn’t have it anymore. Pre-op diagnosis is small bowel obstruction’.

Circulating nurse: ‘Is it?’

The circulating nurse records this diagnosis on the operative record.

Two minutes later, the surgical resident says, ‘I guess you guys don’t have a Belfour?’

The circulating nurse leaves the room and returns with a Belfour retractor.

Circulating nurse: ‘I have a Belfour here if you want me to open it’.

The surgical resident and surgical fellow are talking to each other and do not respond. The scrub nurse and student nurse ask four more times over the next 15 seconds if the surgeons want the Belfour, but never loudly and they get no response. The medical student appears to hear but does not say anything.

Circulating nurse: ‘They want to ignore us. So they’re not going to get the Belfour then’.

There’s no further mention of the Belfour.

In this example, there is a pattern of lack of response to nurses’ questions evident through exchanges about two different topics occurring a few minutes apart. In the first instance, the nurse asks twice about the preoperative diagnosis and gets no response; in the second, the surgeons do not respond to five requests about whether they want a Belfour retractor. The lack of response to the nurses’ questions may reveal the workings of front and back stage concerns on the part of other professionals. The surgical resident may not want to risk being contradicted by the colleague about the choice of retractor. The medical student does not attempt to facilitate relaying the queries to the more senior surgical colleagues, possibly reflecting the trainee’s standing in the OR hierarchy, and possibly also awareness that the more senior surgical team members have heard the nurses’ questions despite the lack of response. The surgeons’ need to concentrate may also be playing a role in the lack of response. An interesting feature of this field note excerpt is that the circulating nurse interprets the silence as deliberate: ‘They want to ignore us’. In this interpretation, we can see an example of the potential interplay between speech and silence, as the nurse’s frustration with the surgical trainees’ non-responses may inhibit future communication.

This example displays two recurrent issues in the data set. The first relates to the audibility of the nurses’ speech (‘The scrub nurse and student nurse ask four more times…*but never loudly*’). We will explore this further in the next section. The second relates to failure of the performative aspect of the nurses’ speech. The performative aspect of speech relates to speech that attempts to accomplish an action, and can ‘fail’ or ‘succeed’ or be more or less efficacious in accomplishing that action. The notion of performative aspects of speech, articulated by Austin (1962), has influenced the notion of language as social action, which is predominant in linguistic anthropology and social theories of language (Bourdieu 1991, Butler 1997, Ahearn 2001), and is frequently analysed in terms of power relations in speech.

We recorded instances of failures to resolve the purpose of the speech acts of all professions in the OR. However, we observed a distinctive patterning of irresolution with regard to nurses’ speech acts. The following example illustrates both the ‘quiet’ volume of the nurses’ speech and the unresolved nature of the nurses’ speech acts. In this example, the nurses try to initiate a pause before the surgery (The surgical pause is a common patient safety protocol in which OR team members review key details before a surgery commences, such as patient name, surgical procedure and site):

Everyone is in place to begin the surgery. The anesthesiologist is chatting with the surgeon.

Circulating nurse: {quiet voice}‘Surgical pause, please’.

Scrub nurse: {repeats, also quiet}‘Surgical pause’.

The anesthesiologist is still talking to the surgeon. The surgical resident repeats ‘pause’ but the surgeon is not paying attention, possibly ignoring, and the resident does not follow up. The scrub nurse with scalpel in hand, and therefore the surgeon’s attention, says again, ‘The surgical pause’. There is another short pause before the resident says, ‘Oh, what do you want to know about his guy? We don’t know what we’re going to do. We may not do anything. How’s that?’

Anesthesiologist: ‘He has no allergies’.

Surgeon: ‘Oh good’. {sarcastic undertone}

Anesthesia resident: ‘Has he gotten some - ?’

Anesthesiologist: ‘He’s gotten some antibiotics’.

Surgeon: ‘How’s that? Can we start?’

Anesthesiologist: ‘Ah, okay’.

Surgeon looks to scrub nurse, who hands the first instrument.

The nurses’ speech act in requesting or reminding the surgeon to complete the surgical pause is somewhat ineffectual in terms of occasioning a complete pause. A partial surgical pause takes place, with the anaesthesia team confirming some information while surgical team members respond with sarcasm or non-participation. The silence of the surgeon could be interpreted as a reflection of resistance to this form of institutional protocol, or an assertion of traditional surgical power. A surgeon participating in our study identified this type of behaviour as a defensive rather than assertive posture, linking the opposition of some surgeons to preoperative communication protocols to an insecure stance in relation to a slowly eroding notion of surgeon autonomy and an emerging conception of surgeons as part of a ‘team’.

For Bourdieu, the efficacy of a speech act is contingent on the authority of the speaker, and that authority derives from institutional power. The power of speech to act, the power of the performative, does not reside in the words that are spoken, but derives from the social power of the person who utters them. Butler (1997) critiques what she refers to as Bourdieu’s static notion of authority, however. While silence and speech are underlined by power dynamics, the authority of speakers is somewhat variable and contingent, assigned through formal and official discourses but also through various, diffuse and tacit processes. The nurses in the above example have institutionally sanctioned authority in initiating a surgical pause. In this sense, the action of the surgeon may be seen as a form of silence deployed to resist the institutional practice of the pause.

In fact, power struggles between nurses and surgeons are often most explicit in interactions over nurses’ power and positioning as sanctioned supervisors of institutional ‘rules’. In the following exchange, a nurse wants to verify whether a patient has been ‘prepped’ (prepared) for surgery. The nurse appears to want further information from the surgeon, which he in turn appears reluctant to provide:

The surgeon tells the two surgical residents to scrub while he preps the patient. He tells them the name of the sterile solution he will use. The solution is clear and invisible when it dries on the patient’s skin.

A short time later, the circulating nurse looks at the surgical site and asks the surgeon if he has prepped the patient.

Surgeon: ‘Yes’.

Nurse: ‘Are you sure?’

Surgeon: ‘Yes’.

Nurse: ‘Really?’

Surgeon: ‘Yes’.

Nurse: ‘Okay’.

The nurse’s doubtful tone and raised eyebrows suggest that she is not entirely convinced.

The surgeon does not provide the nurse with details about the type of prep solution that he used, only reaffirming that the patient had been prepped. The back-and-forth exchange suggests a challenge on the part of the nurse and resistance from the surgeon to being questioned. Many of the instances we recorded of nurses having difficulty in obtaining responses or effecting action relate to brief, seemingly mundane, skirmishes that occur in domains of nursing responsibility, including monitoring sterility in the OR, the instrument count and the surgical pause.

### Speaking quietly

In the example of the surgical pause, the nurses speak quietly despite having institutional authority. To the extent that it plays a role in the lack of success of a speech act, speaking quietly may be perceived as a symptom of the traditional view of silence as a passive or quiescent stance. Nurses were recorded as speaking quietly several times in the preceding field note excerpts. In fact, our observations suggest that nurses frequently use repetition of a question, rather than increasing speaking volume, in order to draw out answers to their queries. Also, it is not only that the nurses speak quietly, but also that there is often a contrast in their volume compared to that of surgeons, who will often speak loudly.

This led us to pose the question, ‘How loud can a nurse speak?’ What happens when a nurse increases speaking volume or speaks in a more insistent or assertive tone? How will the nurse be perceived by colleagues from his/her own and other professions? We observed instances of surgeons describing a nurse who was actively monitoring sterility in the OR as a ‘drill sergeant’, or joking about nurses ‘losing it’. We noticed how we, as observers, would pick up on these constructions of nurses at times in our field notes: for example, a nurse who asked a surgeon to change his gloves several times before he complied was described as being ‘agitated’. Is a quiet tone expected of nurses, such that when they do speak more loudly we perceive their tone as problematic? Also, while we often recorded the volume of speech when it was ‘quiet’, we were less likely to describe someone as speaking ‘loudly’. However, approaching the data with a view to the dynamics of silence and power raises several questions. Does loud speech co-create quiet speech? Is loud speech less evident to us because it appears natural; and are we as researchers attuned to the invisible processes of naturalization through which power operates? Most importantly, why are some speakers hesitant, tense, reticent and not entirely audible, while others are confident, at ease, gregarious and perfectly audible, if not in fact loud?

There are examples of quiet tone that seem to suggest silence as structured in the sense of Bourdieu’s (1977) concept of ‘habitus’. Levels of constraint in speech, including tension in speaking, self-censorship and frequent self-correction are aspects of an embodied sense of the (subordinate) place that one occupies in a social space (Bourdieu 1991). In the following example, there appears to be a level of tension felt by the scrub nurse in speaking, such that she repeatedly performs a complicated physical manoeuvre, rather than giving a brief oral instruction to the surgical team, which the surgeon repeatedly invites her to provide:

This communication event takes place over a 45 minute period. The staff surgeon keeps asking the scrub nurse for ‘burning forceps’, but often he hasn’t handed them back to her. Instead he’s placed them on a rubber mat on the patient’s chest. To retrieve them and hand them to the surgeon when he next needs them, the scrub nurse has to step down off her stool, reach around the surgical resident who is standing to her right, come back up on to the stool, and hand them across the patient’s abdomen to the surgeon. The surgeon notices this and says, ‘Just tell me it’s up’ and then ‘We’ll try to remember to pass it back to you’. This happens multiple times, however, with the scrub nurse stepping down and reaching and the surgeon repeating, ‘Just tell me it’s up!’ The scrub nurse looks sort of bewildered. Once she very quietly says, ‘Up,’ but the next time she reaches for it instead. There is no strong emotion in the surgeon’s tone as he repeats the instruction over and over.

It is not clear what the nurse’s reasons were for not wanting to tell the surgeon that the instrument was ‘up’. Regardless, she does not appear to want to speak and the recurring exchange between surgeon and scrub nurse is difficult to explain in the context of what is happening at the time in the OR. From a critical perspective, such examples indicate the necessity to theorize beyond the immediate situational context. In particular, this example is suggestive of a ‘structured disposition’ (Bourdieu 1977) to be silent. The silence we see in this case may reflect an actualization of structured power dynamics; those social, historical, cultural and institutional factors that are reproduced on a daily basis. The silence in this example seems to demonstrate what poststructural theorists have long argued is the most subtle and effective form of power – not the ability to coerce, but power’s ability to produce and create; to shape subjectivities and conceptions of who we are.

Our research supports the finding of other OR ethnographies that the need for nurses to demonstrate competence, often through the ability silently to anticipate surgeons’ needs and preferences, can impede interprofessional communication (Riley & Manias 2005, 2006, Gillespie *et al.* 2007). Nurses often try to resolve problems in a ‘back stage’ manner, at times, no doubt, to avoid bothering surgeons, but at other times to avoid revealing uncertainties and appearing inadequate. The OR is a space characterized by the performance, surveillance and judgment of knowledge and competence. These are processes of power that clearly produce silence and constrained communication. Our research contributes to deepening the understanding of such silencing processes. After examining constraint in naturally occurring communication, we suggest that there is a general reticence that pervades much nurse–physician communication in the OR. Furthermore, the quiet and hesitant tone often evident in nurses’ speech may in part reflect (and reproduce) a social practice in which a nurse who speaks and acts assertively risks losing legitimacy.

However, unlike other research, we also consider silences on the part of other OR professionals, and suggest that silences on the part of nurses and others can be expressive rather than inexpressive, strategic rather than simply defensive. Nurses often use forms of silence to achieve objectives and communicate. Furthermore, our analysis points not only to how individuals exercise power in the OR setting, but also to social and structural aspects of power; for example, silences may reflect predispositions or internalized factors resulting from broader institutional power relations.

We have used critical theories of the role of silence in communication for insights into instances of silence and constrained communication in the OR. These include theoretical approaches emphasizing the need to consider the role of social relations that extend beyond the immediate setting (Bourdieu 1996, Poland & Pederson 1998). We have drawn on the studies of Gal (1991), Glenn (2004),and Brown (2005) to consider how nurses may find space within silence to achieve goals and objectives. Theories of the performative aspects of speech (Austin 1962, Bourdieu 1991, Butler 1997) help delineate what happens when nurses do speak. Recurring instances of non-response and inefficacious speech acts convey strongly the difficulties nurses encounter in communication in the OR, but also areas of daily conflict and negotiation over how communication will take place in the interprofessional setting of the OR.

## Conclusion

Our findings have implications for policy and practice to promote safety in the OR. Policies such as the ‘surgical pause’ tend to focus on speech and speaking. Initiatives to encourage people to talk are important but cannot ignore the complexity of the spectrum of speech and silence, and how speech and silence interact and shape each other. Attention to the complexity of silence in the OR is also essential in the context of increasing movements in health care to ‘foster’ collaboration and ‘improve’ communication in clinical team settings. This ethnography of silence contributes a more nuanced view of interprofessional communication to counter an often tacit assumption that communication proceeds only through explicit, cross-checking, performative speech. Awareness of these nuances and complexities may help nurses and other professionals learn to interpret the multiple modalities and strategies of communication at play in the OR.
